# Toxicity of Arsenic (III) on Isolated Liver Mitochondria: A New Mechanistic Approach

**Published:** 2013

**Authors:** Mir-Jamal Hosseini, Fatemeh Shaki, Mahmoud Ghazi-Khansari, Jalal Pourahmad

**Affiliations:** a *Faculty of Pharmacy, Shahid Beheshti University of Medical Sciences, Tehran, Iran.*; b *Department of Pharmacology and Toxicology, School of Pharmacy, Zanjan University of Medical Sciences, Zanjan, Iran. *; c *Student Research Committee, School of Pharmacy, Shahid Beheshti University of Medical Sciences, Tehran, Iran. *; d *Department of Pharmacology and Toxicology, School of Pharmacy, Mazandaran University of Medical Sciences, Sari, Iran.*; e *Department of Pharmacology, School of Medicine, Tehran University of Medical Sciences,Tehran, Iran.*

**Keywords:** Arsenic (III), mitochondria, Complex I, Complex II, Reactive Oxygen Species

## Abstract

*Arsenic *exposure mainly through food and water has been shown to be associated with increased incidence of numerous cancers and non-cancer harmful health. It is also used in cancer chemotherapy and treatment of several cancer types due to its apoptogenic effects in the various cancer and normal cell lines. We have already reported that liver is the storage site and important target organ in As (III) toxicity and recently, it has been suggested that hepatic toxicity of arsenic could be resulted from impairment of the liver mitochondria. In this study, interaction of As (III) with freshly isolated rat mitochondria was investigated. We determined different mitochondrial toxicity factors as well as mitochondrial sources of ROS formation using specific substrates and inhibitors following addition of As (III) to the mitochondria. Our results showed that arsenic (III) increased mitochondrial ROS formation, lipid peroxidation and mitochondrial membrane potential collapse, cytochrome c release and mitochondrial swelling in a concentration dependent manner. Addition of As (III) in to the isolated mitochondria, inhibited complexes I and II leading to disruption of mitochondrial electron transfer chain, decreased mitochondrial ATP content and ROS formation.

## Introduction

Arsenic is an environmental potent toxic metal and naturally occurring element which is released both from human activities and also naturally from the Earth’s crust. It is widely distributed in the soil, water and food, and is very harmful to the environment and to humans because of *in-vivo *accumulation in liver, kidney and other tissues (1).On the other hand, arsenic trioxide (As III) is used in cancer chemotherapy and treatment of several type cancer including chronic myeloid leukemia (CML), lymphoma, esophageal cancer and particularly acute promyelocytic leukemia (APL) probably due to apoptosis induction (2). Hence, arsenic exposure mainly through food and water was shown to be associated with increased incidence of numerous cancers (*e.g*., skin, bladder, liver, lung and urinary) and non-cancer harmful disorders (*e.g*., skin lesions, peripheral vascular disease, cardiovascular disease, and diabetes mellitus) mainly due to consumption of arsenic contaminated drinking water (3).Epidemiologic and experimental published reports from the International Agency for Research on Cancer (IARC) concluded that arsenic is a human carcinogen (4). In addition, previous studies showed that liver is one of the most important targets for As (III) toxicity (5, 6).

 It has already been reported that arsenic induces a loss of mitochondrial membrane potential and induces the generation of reactive oxygen species (ROS) and lipid peroxidation as primary mechanisms for toxicity (7). On the other hand, it was reported that As (III) activates caspase cascade signaling and induces cell apoptosis through induction of mitochondria permeability transition (MMP), alteration of mitochondria function proteins (Bcl-2, Bax, and cytochrome c), and marked increase in caspase activities resulting in oxidative stress and activation of the endoplasmic reticulum (ER) stress pathway (3, 8). In addition, pretreatment of As (III) with N-acetylcysteine (NAC, 0.5 mM) dramatically suppressed the increases in reactive oxygen species (ROS), lipid peroxidation, ER stress, caspase cascade activity, and apoptosis in As_2_O_3_ (10 μM)-treated myoblasts (8). Recently, it has been suggested that hepatic toxicity of arsenic could be resulted from impairment of the liver mitochondria and it is suggested that alteration of mitochondrial structure is an early event of As (III)-induced apoptosis in esophageal carcinoma cells (5). Experiments performed by Bustamante et al revealed that As (III) induces both apoptosis and necrosis when administered in vitro in different cellular systems through failure of mitochondrial electron transport system, induction of mitochondrial permeability transition and subsequent release of cytochrome c (9).

Recent publications suggested that there are several possible sources responsible for elevation of ROS levels including interactions with the respiratory chain complexes; oxidation of As (III) to As (V) is evident in liver metabolism as well as the release of iron from ferritin by trivalent arsenic species. Furthermore, interactions of arsenic with cellular antioxidant mechanisms, in particular a decrease in glutathione levels and finally the disturbance of DNA repair systems, contribute to increased levels of oxidative damage in cells (10, 11).There is strong evidence linking arsenic induced oxidative stress and endothelial inflammation, which is a hallmark of atherosclerosis (12). 

It has been estimated that 1–2% of oxygen consumed systemically is converted to reactive oxygen by mitochondria, which is a major target organelle for oxidative damage and more importantly mitochondria is responsible for production of 60 80% of H_2_O_2_ in cells under normal condition (13). Mitochondrial H_2_O_2_ generation can be increased in pathological conditions associated with inhibition of mitochondrial electron transfer chain complexes (14). Since mitochondria does not have the complexity of the isolated cells, which have both the barrier of membrane permeability and an active metabolizing system (15). Due to the key role that mitochondria play in As (III) toxicity; we planned to investigate the mitochondriotoxicity mechanisms of As (III) in isolated rat liver mitochondria. Since, all the mitochondrial pathways involved in arsenicinduced mitochondrial damage have not yet been completely understood; we postulated that arsenic liver toxicity is result of its disruptive effect on liver mitochondrial respiratory chain and subsequently induction of ROS formation and lipid peroxidation. Our results supported the hypothesis that arsenic targets mitochondria directly, particularly complex I of the mitochondrial respiratory chain, it will cause mitochondrial instability and decrease of ΔΨ that facilitates permeability transition and necrotic cell death.

## Experimental

*Materials*

Sodium arsenite (NaAsO_2_) and other chemicals were purchased from Sigma-Aldrich Chemical Co. (St. Louis, MO, USA). All chemicals were of analytical grade, HPLC grade or the best pharmaceutical grade.

*Preparation of mitochondria*

All experimental procedures were conducted according to the ethical standards and protocols approved by the Committee of Animal Experimentation of Shahid Beheshti University of Medical Sciences, Tehran, Iran. All efforts were made to minimize the number of animals used and their suffering according to protocols issued by committee of ethics, Faculty of pharmacy, Shahid Beheshti university of medical sciences. Mitochondria were prepared from Wistar rat’s liver using differential centrifugation (16). The animals were sacrificed by decapitation and their liver were quickly removed and placed in a beaker, the tissues were minced and homogenized with glass handheld homogenizer in icecold mitochondria isolation medium (0.225 M D-manitol, 75 mM sucrose, and 0.2 mM EDTA, pH 7.4). The nuclei and broken cell debris were sedimented by centrifuging at 1500×*g *for 10 min at 4ºC and the pellet was discarded. The supernatant then was subjected to a further centrifugation at 10,000×*g *for 10 min and the superior layer was carefully discarded. The mitochondrial pellet was washed by gently suspending in the isolation medium and centrifuged again at 10,000×*g* for 10min. Finally the mitochondria pellet were suspended in Tris buffer containing (0.05 M Tris-HCl, 0.25 M sucrose, 20 Mm KCl, 2.0 mM MgCl_2_, and 1.0 mM Na_2_HPO_4_, pH 7.4)at 4◦C . Protein concentrations were determined by the Bradford method using BSA as a standard (17). Mitochondria were prepared fresh for each experiment and used within 4 h of isolation and all steps were strictly operated on ice to guarantee the isolation of high-quality mitochondrial preparation. The concentrations of Arsenic III (50, 100, 200 μM) were partly chosen based on our previous study (6) and Isolated mitochondria obtained in this way were incubated in Tris buffer with 3 mentioned concentrations of arsenic at 30 °C for 1 h.

*Quantification of mitochondrial ROS level by flow cytometry*

The mitochondrial ROS measurement was performed by flow cytometry (Partec, Deutschland) using DCFH-DA in the interval times of 5, 30 and 60 min (18). Signals were obtained using a 530-nm band pass filter (FL-1 channel). Each determination is based on mean fluorescence intensity of 15,000 counts.

*Evaluation of complexes I and III on arsenicinduced H*_2_ *O*_2_ *formation*

Role of complex I and III in arsenic -induced H_2_O_2_ production were determined spectroflorimetrically using homovanillic acid and horseradish peroxidase at 312 nm excitation and 420 nm emission wave lengths with and without the presence of 50U/ml of superoxide dismutase (19). Rotenone (2 μM) and antimycin A (2 μM) as mitochondrial respiratory complexes inhibitors were also added in the reaction in some groups.

*Complex II activity assay using MTT test*

The activity of mitochondrial complex II was determined by measurement of reduction of MTT as explained by Zhao *et al. *(20). 

*Measurement of Lipid peroxidation*

The content of MDA was determined using the method of Zhang et al. 2008 in mitochondrial samples (0.5 mg protein/mL). The amount of MDA formed in each of the samples was assessed by measuring the absorbance of the supernatant at 532 nm with an ELIZA reader (Tecan, Rainbow Thermo, Austria). Tetramethoxypropane (TEP) was used as standard and MDA content was expressed as nmol/mg protein (21).

*Measurement of GSH content*

GSH content was determined using DTNB as indicator by spectrophotometric method in isolated mitochondria. The yellow color developed was read at 412 nm using a spectrophotometer (UV-1601 PC, Shimadzu, Japan). GSH content was expressed as μg/mg protein (22).

*Determination of the mitochondrial membrane potential (MMP)*

Mitochondrial membrane potential was measured by determination of mitochondrial uptake of cationic fluorescence probe rhodamine123 (23).The rhodamine123 fluorescence was monitored using Schimadzou RF-5000U fluorescence spectrophotometer at the excitation and emission wavelengths of 490 nm and 535 nm, respectively

*Determination of mitochondrial swelling*

Mitochondrial swelling in isolated mitochondria (0.5 mg protein/ml) was assessed by determination of changes in light scattering as monitored spectrophotometrically at 540 nm(30°C) even 10 min time intervals with an ELISA reader (Tecan, Rainbow Thermo, Austria) as described (20).

*Measurement of cytochrome-c oxidase activity and assessment of outer mitochondrial membrane damage*

Both factors were evaluated using cytochrome-*c *oxidase assay kit (Sigma, St. Louis, MO) according to the manufacturer’s protocol. The decrease in absorbance at 550 nm is related to oxidation of ferrocytochrome-*c *by cytochrome-*c *oxidase. Cytochrome-*c *oxidase activities were calculated and normalized for the amount of protein per reaction, and results were expressed as units per milligram mitochondrial protein. Mitochondrial outer membrane integrity was assessed by measuring cytochrome-*c *oxidase activity of mitochondria in the presence or absence of the detergent, *n*-dodecyl *β*-D-maltoside. The mitochondrial outer membrane damage was assayed from the ratio of cytochrome-*c *oxidase activity in the presence and absence of detergent n-dodecyl *β*-D-maltoside.

*Assay of ATP and ATP/ADP ratio*

The ATP and ATP/ADP ratio levels were measured by luciferase enzyme (24). Bioluminescence intensity was measured using Sirius tube luminometer (Berthold Detection System, Germany).

*Cytochrome c release assay*

The concentration of cytochrome *c *was determined by using the QAsntikine Rat/Mouse Cytochrome*c *Immunoassay kit provided by R & D Systems, Inc. (Minneapolis, Minn) and the optical density of each well was determined by the aforementioned microplate spectrophotometer set to 450 nm.

*Statistical Analysis*

Results are presented as means ± SD. All statistical analyses were performed using the SPSS software, version 17. Assays were performed in triplicate and the mean was used for statistical analysis. Statistical significance was determined using either the one-way ANOVA test, followed by the posthoc Tukey test or two-way ANOVA test, followed by the posthoc bonferroni test. Statistical significance was set at p < 0.05.

## Results

**Figure 1:**
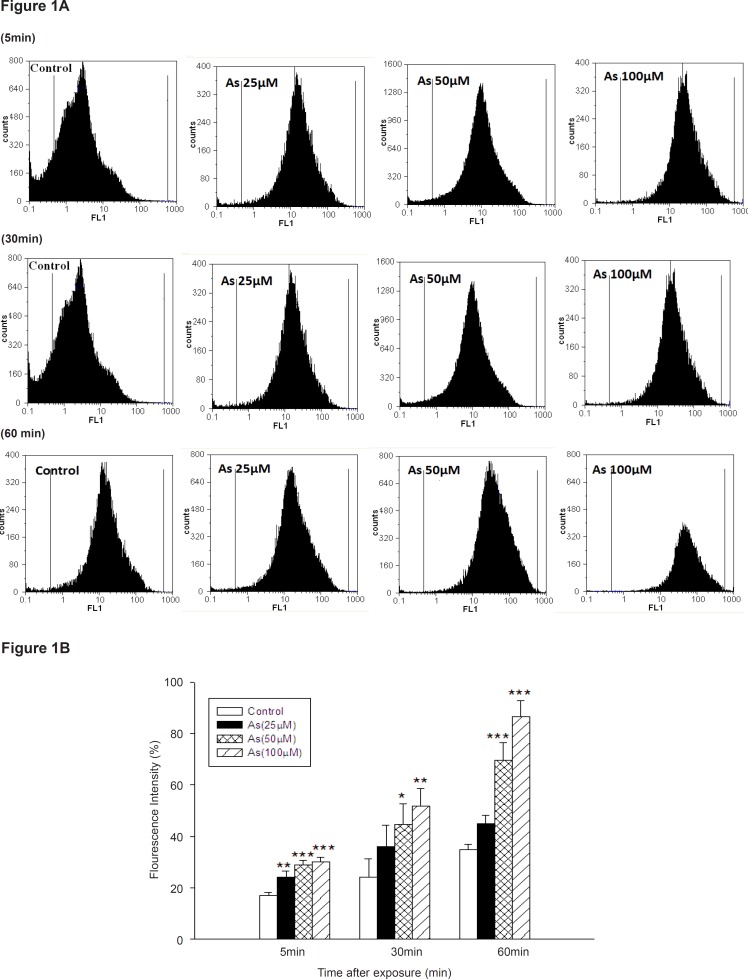
ROS formation in arsenic treated mitochondria. ROS formation after addition of various concentrations of arsenic (0, 25, 50 and 100 μM) at interval a (5min after addition), b (30 min after addition) and c (60min after addition).d (summary of ROS formation), ROS formation was determined by flow cytometry using DCF-DA as described in Materials and methods. FL1: the fluorescence intensity of DCF. *p < 0.05; **p < 0.01; ***p < 0.001 compared with control mitochondria.

As shown in Figure 1, different concentrations of As (III) induced significant ROS formation in isolated liver mitochondria. 

Our study showed that As (III) could induce ROS production in concentration and time dependent manner. But, low concentration of sodium arsenite (10 μM) did not significantly increase ROS generation until 60 min (Data not shown), whereas rate of ROS formation significantly increased at 30 and 60 min, following exposure to sodium arsenite (50 μM).A more substantial increase in mitochondrial ROS formation was observed in highest concentration of As (100 μM) at 30 and 60 min following addition (p < 0.05).

**Figure 2:**
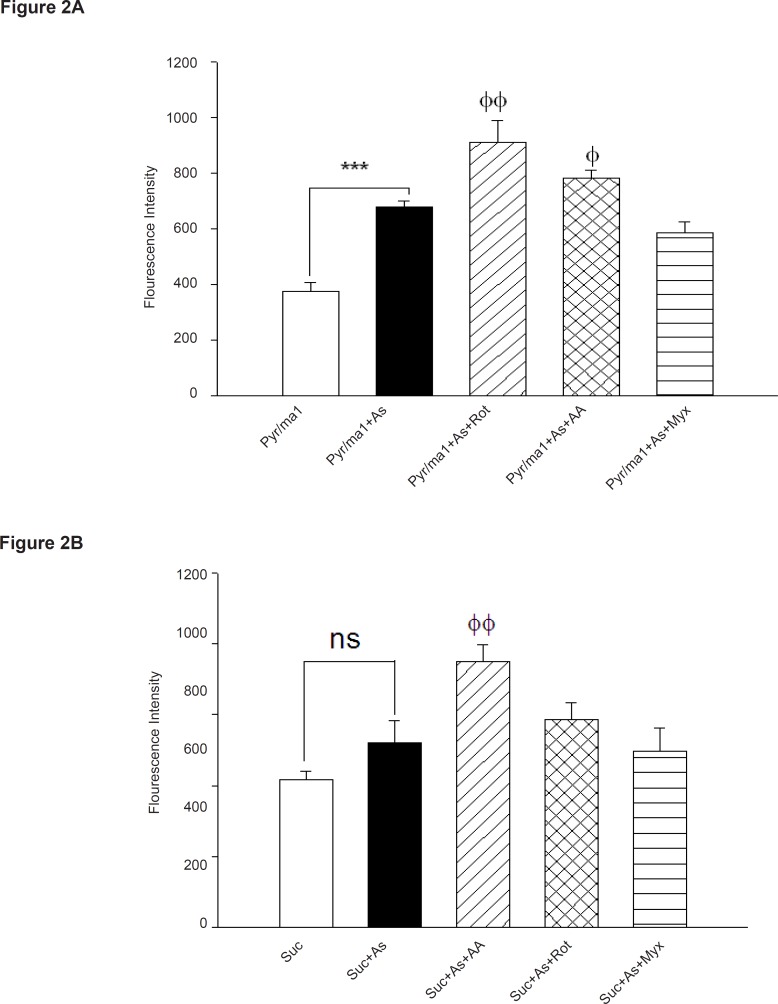
Influence of respiratory substrates and inhibitors of complex I and III on arsenic -induced ROS formation in isolated liver

 To assess the involvement of complex I and III on arsenic induced- ROS production in liver mitochondria, we used a fluorescent dye/horseradish peroxidase detecting system employing the peroxidase substrate homovanilic acid (Figure 2). 

mitochondria. The measurements were performed with the homovanilic acid/horseradish peroxidase method, as described in Materials

and methods.

A: ROS formation was measured in the presence of: pyruvate 2.5 mM +malate 2.5 mM alone (pyr/mal), pyruvate /malate +arsenic

50 μM (pyr /mal +As), pyruvate/malate + arsenic 50 μM +Antimycin A 2 μM (pyr/mal +As+AA), pyruvate /malate+ arsenic 50 μM +

rotenone 2μM (pyr /mal+As+Rot), pyruvate /malate+ arsenic 50μM + myxothiazole 5 μM (pyr /mal+As+Myx), Values represented as

mean±SD (n=3). *p < 0.05; **p < 0.01; ***p<0.001 compared with Pyr/mal group, ^Φ^p < 0.05; ^ΦΦ^p < 0.01^ΦΦΦ^p < 0.001, compared with

pyr/mal + As groups.

B: ROS formation was measured in the presence of: succinate 5mM alone (Suc), succinate+ arsenic 50 μM (Suc+ As), succinate+

arsenic 50 μM + Antimycin A 2 μM (Suc+ As +AA), succinate+ arsenic 50 μM + rotenone 2μM (Suc+ As +Rot), succinate+ arsenic 50

μM + myxothiazole 5 μM (Suc+ As +Myx).Values represented as mean±SD (n=3). *p < 0.05; **p < 0.01; ***p < 0.001 compared with

Suc group, ^Φ^p < 0.05; ^ΦΦ^p < 0.01^ΦΦΦ^p < 0.001, compared with Suc+As groups.

As demonstrated in Figure 2, As (III) produced significant amounts of ROS in isolated liver mitochondria in the presence of the NAD-linked substrates, malate and pyruvate (pyr/mal), even greater than that was observed in the presence of Complex III-linked substrates succinate. Whereas, addition of the respiratory Complex III inhibitor Antimycin A again increased higher H_2_O_2_ production in isolated liver mitochondria, but myxothiazol (semiquinone formation inhibitor) did not show any significant effect in H_2_O_2_ production (p > 0.05). Maximum level of H_2_O_2_ production achieved following addition of rotenone (specific inhibitor of complex I). On the other hand, our results showed the highest rate of protection against H_2_O_2_ production

achieved in presence of SOD.

**Figure 3:**
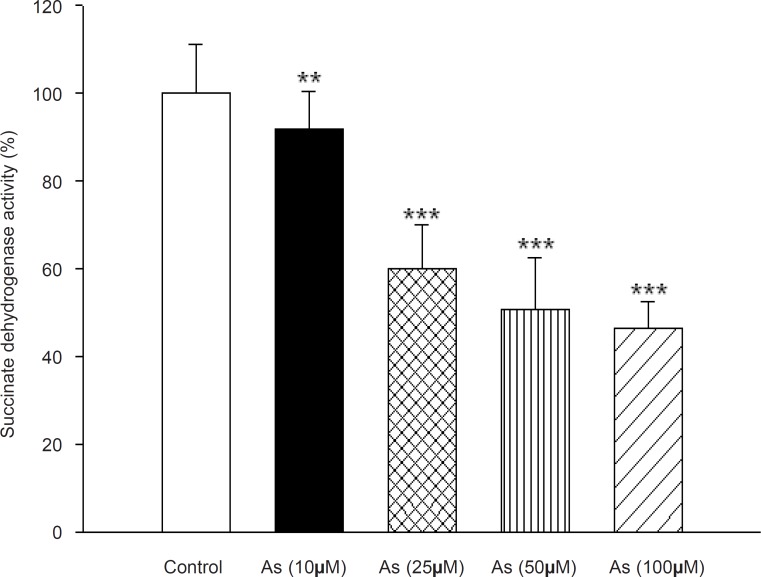
Effect of arsenic on Succinate dehydrogenase (complex II) activity. Succinate dehydrogenase activity was measured using MTT dye as described in Materials and methods. Liver mitochondria (0.5 mg/mL) were incubated for 1 h with various concentrations of arsenic (0, 10, 25, 50 and 100 μM). Values represented as mean±SD (n = 3). *p < 0.05; **p < 0.01; ***p < 0.001 compared with control mitochondria.

 Succinate dehydrogenase activity (complex II) was assessed by MTT reduction after 1h incubation of mitochondria with different concentrations of sodium arsenite (25, 50 and 100 μM). Figure 3 showed a significant decrease in the mitochondrial reduction of MTT to formazan (p < 0.05) .On the other hand, Cytochrome-*c* oxidase activity (complex IV), one of the most important enzymes in the mitochondrial respiratory chain, was also determined in the isolated liver mitochondria following addition of As (III) (p > 0.05). Our results showed no significant change in activity of this enzyme during the incubation time.

**Figure 4:**
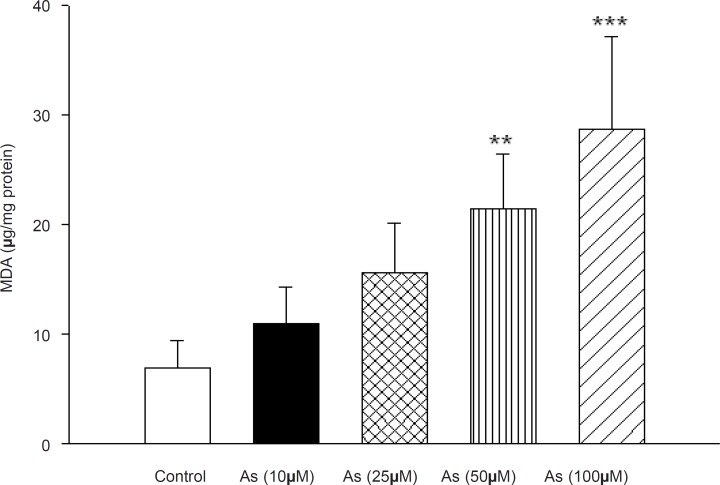
Effect of arsenic on lipid peroxidation in liver mitochondria. MDA formation was measured using thiobarbituric acid reactive substances assay as described in Materials and methods. Liver mitochondria (0.5 mg/mL) were incubated for 1h with various concentrations of arsenic (0,10, 25, 50 and 100 μM). Values represented as mean±SD (n = 3). *p < 0.05; **p < 0.01; ***p < 0.001 compared with control mitochondria.

 Besides, cyanide addition to arsenic treated mitochondrial showed the same inhibition of cytochrome c oxidase activity similar as was recorded with cyanide, alone (Data not shown). Lipid peroxidation was also assayed in As (III) treated in rat liver mitochondria that following the addition of different concentrations of arsenite (10, 25, 50 and 100 μM). As shown in Figure 4 the amount of MDA formation(marker of lipid peroxidation) in mitochondria were11± 3.3, 15.6 ± 4.5, 21.4 ± 9.6 and 28.7 ± 8.5 μg MDA/mg protein at 10, 25, 50 and 100 μM arsenic concentrations, respectively, whereas that of control group was 6.9 ± 2.4MDA/mg protein. Our data shows that the mitochondrial MDA was only significantly increased in high concentration of arsenic (50 and 100 μM).

**Figure 5:**
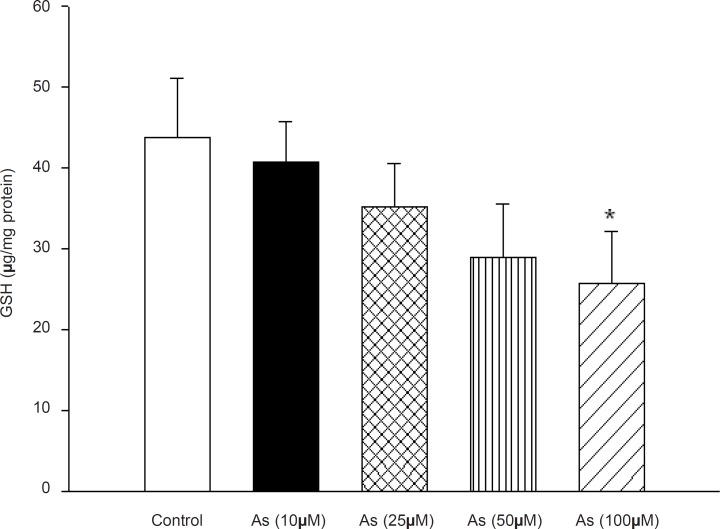
Effect of arsenic on mitochondrial GSH content. Liver mitochondria (0.5 mg/mL) were incubated for 1h with various concentrations of arsenic (0, 10, 25, 50 and 100 μM). Values represented as mean±SD (n = 3). *p < 0.05; **p < 0.01; ***p < 0.001 compared with control mitochondria.

Due to our findings which strongly support a key role for arsenic in mitochondrial H_2_O_2_generation and lipid peroxidation, we decided to determine the possible effect of this metal on the mitochondrial antioxidant systems. Mitochondrial GSH, a very important antioxidant defense against ROS formation was measured spectrophotometerically using DTNB as indicator in isolated mitochondria after 1 h exposure to arsenic. GSH levels decreased down to 41±5, 35±5, 28±5and 26±7 (μg/mg protein) respectively. Compared to control mitochondria 44±7 (μg /mg protein), only in high concentration of arsenite (100 μM), there was a significant decrease in mitochondrial GSH content compared to control mitochondria only in the highest As (III) concentration applied (p < 0.05) (Figure 5).

The effect of arsenic on mitochondrial membrane potential (MMP) measured by Rh123 staining test, the MMP significantly decreased in As (III) treated mitochondrial in concentration and time related manner (p < 0.05). On the other hand pretreatment of Cs A (1 μM), an inhibitor of MPT pore, and BHT (20 μM), an antioxidant, significantly inhibited collapse of MMP induced by 50 μM of Arsenic(p < 0.05) (Table 1).

**Table 1:** The effect of arsenic (As) on the mitochondrial membrane potential (MMP) in liver mitochondria. MMP was measured by rhodamin 123 as described in Materials and methods. a) The effect of arsenic (0, 50, 100 and 200 μM) on the mitochondrial membrane potential in liver mitochondria b) The effect of cyclosporine A (1 μM) and BHT (20 μM) on arsenic-induce MMP collapse. Values represented as mean ± SD (n=3). *p < 0.05; **p < 0.01; ***p < 0.001 compared with control mitochondria (a) and AS (100 μM) treated mitochondria (b).

**Percent decrease of MMP (%)**							
	**5 min**	**10 min**	**20 min**	**30 min**	**40 min**	**50 min**	**60 min**
Control	0	5±1	10±2	28±3	34±3	35±4	37±5
As (10μm)	6±1	35±3***	60±3***	67±4***	76±5***	55±3***	58±6***
As (25μm)	7±1	49±2***	69±4***	73±5***	77±2***	83±7***	80±4***
As (50μm)	13±2	50±5***	70±4***	75±4***	84±3***	94±6***	94±7***
As (100μm)	27±2	71±4***	65±2***	76±3***	86±3***	97±6***	105±9***
As (50μm) +CSA	3±2	6±1###	10±1###	29±1###	31±5###	40±3###	53±3###
As (50μm) +BHT	5±3	4±4###	21±3###	28±6###	42±7###	49±5###	50±8###
Cacl_2_	60±6***	81±3***	82±5***	96±5***	100±4***	101±3***	100±1***

In addition, mitochondrial swelling, an indicator of mitochondrial membrane permeability, was monitored with changes of absorbance at 540 nm (A_540_). A decrease in absorbance indicates an increase in mitochondrial swelling. Our results showed that As (III) induced mitochondrial swelling is concentration and time dependent. Furthermore, Cs A prevented As (III) induced mitochondrial swelling exposed to 50 μM arsenic (p < 0.05) (Table 2).

**Table 2:** The effect of arsenic (As) on the mitochondrial swelling in liver mitochondria. Mitochondrial swelling was measured by determination of absorbance at 540 nm as described in Materials and methods. Values represented as mean ± SD (n=3). *p < 0.05; **p < 0.01; ***p < 0.001 compared with control mitochondria and ^$^p < 0.05;^$$^p < 0.01;^ $$$^p < 0.001 compared with AS (100 μM) treated mitochondria.

**Mitochondrial Swelling percent (%)**							
	**5 min**	**10 min**	**20 min**	**30 min**	**40 min**	**50 min**	**60 min**
Control	0	2 ± 1	7 ± 2	10 ± 3	13 ± 3	21 ± 4	34 ± 3
As (10μm)	2 ± 1	4 ± 1	7 ± 1	10 ± 2	21 ± 3***	29 ± 4***	35 ± 5***
As (25μm)	2 ± 1	13 ± 2***	15 ± 2***	24 ± 2***	27 ± 3***	33 ± 3***	36 ± 4***
As (50μm)	1 ± 1	16 ± 2***	32 ± 2***	38 ± 3***	44 ± 2***	46 ± 3***	58 ± 3***
As (100μm)	27±2	20 ± 2***	34 ± 2***	43 ± 3***	46 ± 3***	52 ± 3***	59 ± 4***
As (50μm) +C_S_A	3 ± 2	8 ± 2###	10±1###	12 ± 3###	26 ± 2###	27 ± 3###	28 ± 2###
As (50μm) +BHT	3 ± 2	7 ± 3###	21±3###	11 ± 2###	25 ± 3###	29 ± 5###	30 ± 4###

**Figure 6:**
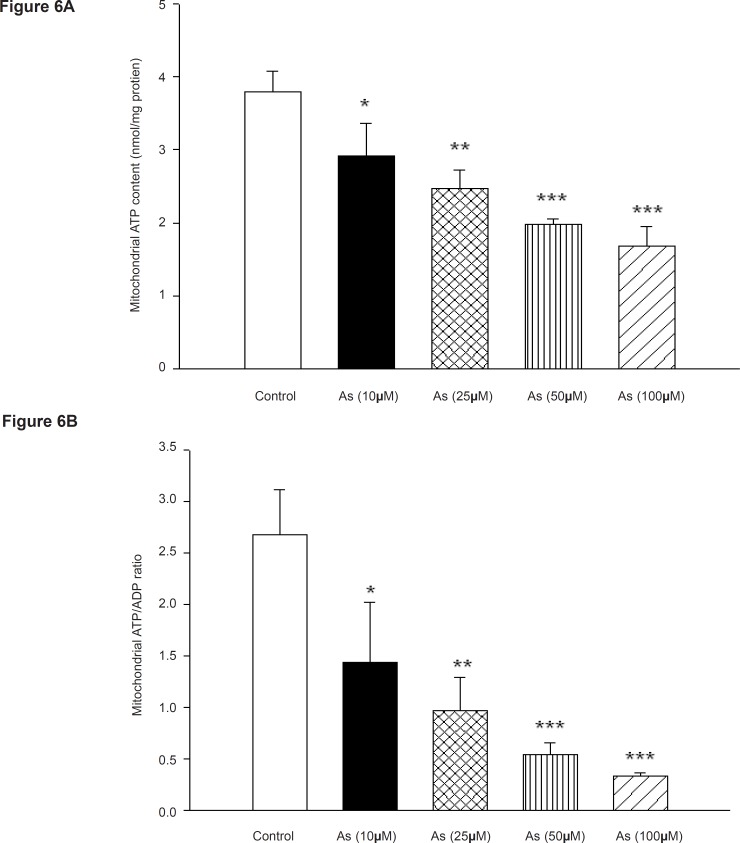
Effect of arsenic on mitochondrial a) ATP level and b) ADP / ATP ratio. Liver mitochondria (0.5 mg/mL) were incubated with various concentrations of arsenic (0, 10, 25, 50 and 100 μM) and ATP level and ATP/ADP ratio were determined using Luciferin/ Luciferase Enzyme System as described in Materials and methods. Values represented as mean±SD (n=3). *p < 0.05; **p < 0.01; ***p < 0.001 compared with control mitochondria

The most important function of mitochondria is the generation of ATP by oxidative phosphorylation, since incubation of mitochondria with arsenic impaired the mitochondrial respiration; we decided to measure the ATP levels and ATP/ADP ratios in isolated rat liver mitochondria following the addition of different concentrations of arsenic. As shown in Figure 6, Mitochondrial ATP levels and ATP/ADP ratio were also decreased in concentration dependent manner. Mitochondrial ATP levels were significantly changed by the AS concentrations of 100 and 200 μM but did not significantly change from control at concentration of 50 μM (Figure 6A). Decline of ATP/ADP ratios in isolated liver mitochondria were significant at As (III) concentrations of 100 and 200 μM (p < 0.05) (Figure 6B). This reduction of mitochondrial ATP content indicates mitochondrial dysfunction leading to decrease in its ability of oxidative phosphorylation. On the other hand fall in the ATP content may exacerbate the ROS formation and lipid peroxidation and finally shifting apoptosis to ultimate necrosis.

**Figure 7:**
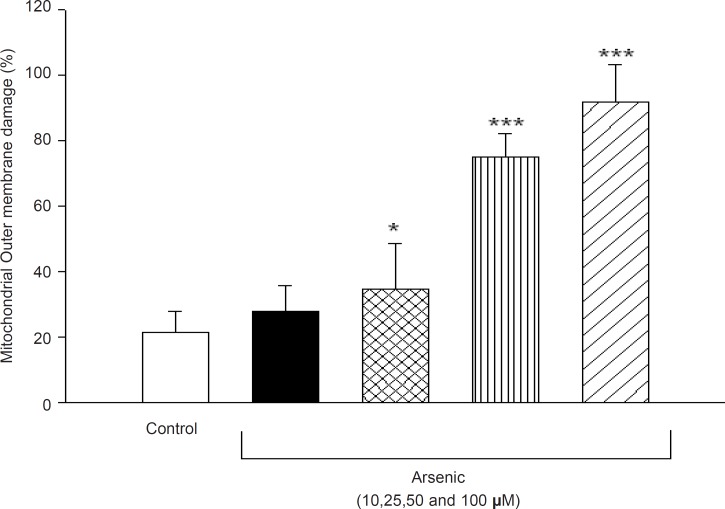
Effect of arsenic on mitochondrial outer membrane integrity. Liver mitochondria (0.5 mg/ML) were incubated for 1h in the presence of different concentration of arsenic (0, 10, 25, 50 and 100 μM) and mitochondrial outer membrane integritywas measured as described in method and materials. Values represented as mean±SD (n=3). *p < 0.05; **p < 0.01; ***p < 0.001 compared with control mitochondria.

Mitochondrial outer membrane integrity was assayed in the isolated liver mitochondrial after 1h incubation to arsenic (10, 25, 50 and 100 μM). So, cytochrome c oxidase activity was measured in the presence and absence of detergent *N*-dodecyl *β*-D-maltoside. This ratio represents the percentage of mitochondrial outer membrane damage. As shown in Figure 7, mitochondrial outer membrane damage was significantly increased in As (III) treated mitochondria in concentration dependent manner (p < 0.05).

**Figure 8:**
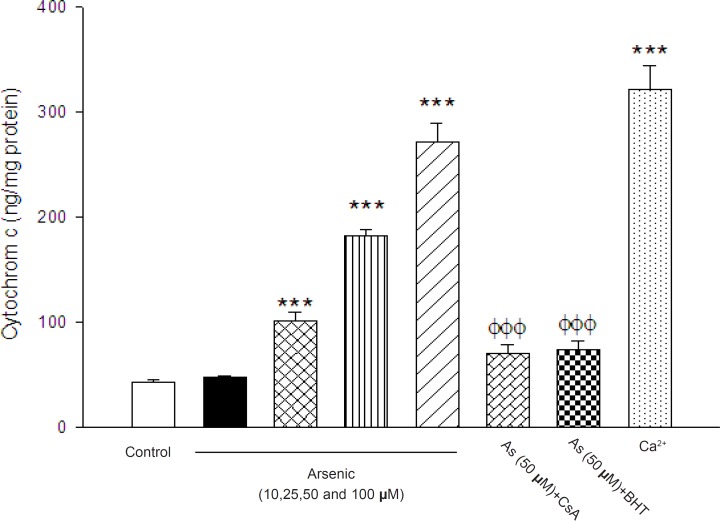
The effect of arsenic (As) on the cytochrome c release. The amount of expelled cytochrome c from mitochondrial fraction was determined using Rat/Mouse Cytochromec ELIZA kit as described in Materials and methods. Values represented as mean±SD (n=3). *p < 0.05; **p < 0.01; ***p < 0.001 compared with control mitochondria and^ Φ^p < 0.05; ^ΦΦ^p < 0.01; ^ΦΦΦ^p < 0.01 compared with As(50 μM) treated mitochondria

Our results show that arsenic significantly caused collapse of the mitochondrial membrane otential and disruption of mitochondrial outer membrane integrity. Therefore, we hypothesized

that arsenic might induce release of cytochrome c from mitochondria into cytosolic medium and

initiated apoptosis signaling. As shown in Figure 8, there were statistically significant differences

in the quantity of cytochrome c in both control and arsenic-treated mitochondria (p < 0.05). We

also found that the cytochrome c release from mitochondrial fraction was increased following treatment with arsenic, suggesting penetration of cytochrome c from the mitochondria into the cytosol in intact cells. Importantly, the pretreatment of arsenic treated mitochondria with the MPT inhibitor CsA and BHT significantly inhibited cytochrome c release, compared to arsenic (50 μM) treated group (p < 0.05), suggesting the key role of oxidative stress in MPT mediated cytochrome c release.

## Discussion

Mitochondria not only play a key role in producing energy in a process called oxidativephosphorylation (OXPHOS), but also a central role in apoptosis, cellular stress response and various genetically inherited diseases (25).

It was found that mitochondria might have a pathogenic role in human disease that typically affects tissues with great energy demand such as liver, kidney, eye, central nervous system, heart and skeletal muscle (26).Defective OXPHOS is known to affect several cellular processes, such as the redox status of the cell and sub-cellular fractions, production of ATP, reactive oxygen species (ROS) formation and induction of apoptosis (26).Mitochondria are also considered as potential targets for environmental pollutants such as metals. Evidence showed that metals induce oxidative stress via mitochondrial dysfunction (18). In this research, we aimed to study the effects of arsenic on mitochondriotoxicity parameters such as swelling, Ψm, enzymatic activity such as succinate dehydrogenase and cytochrome c oxidase using isolated rat liver mitochondria. 

Isolated mitochondria preparations offer a number of advantages over intact cells, particularly by their ability to manipulate access to substrates and alter the local environment (27, 28). Now a days, advances in mitochondrial research and also in medical technology have been a major impetus behind the desire to design and develop new drugs specifically targeting mitochondria for therapeutic benefit in several diseases and also understanding of cellular and molecular mechanisms of xenobiotics (25). Experiments performed by Bustamante et al, revealed that arsenic via release of cytochrome c induces both apoptosis and necrosis when administered in vitro in different cellular systems failure via induction in electron transport system, induction of mitochondrial permeability transition and subsequent swelling of mitochondria (9). Since arsenic, stimulates permeability transition, the induction of MPT was suggested as a key step in apoptosis induced by arsenic in intact APL cells, malignant lymphocytes and carcinoma cell line (2, 29). An our previous studies demonstrated that arsenic could cause increase in ROS formation and also lipid peroxidation and disrupt the MMP in isolated hepatocyte (6, 37).Therefore, it is assumed that mitochondria might be the most important target of arsenic in cells which play important role in arsenic-induced hepatotoxicity. In addition, previous studies in isolated hepatocyte and cell lines showed that mitochondria are involved in apoptosis and release of cytochrome c and aspase activation. On the other hand, there is a strong body of evidence linking arsenic induced oxidative stress and endothelial inflammation, which is the hallmark of atherosclerosis (12, 29). Other studies showed that low levels of arsenite promote endothelial cell tube formation and angiogenesis in both cell culture and in vivo mouse models, nontheless higher doses of arsenic are toxic to endothelial cells and inhibit angiogenesis (30, 31).Therefore, mitochondrial dysfunction are important mechanisms of xenobiotics-induced hepatotoxicity (32). Our data showed that incubation of isolated liver mitochondria with arsenic impaired electron transfer chain that leads to increased ROS formation, lipid peroxidation and ATP depletion. In addition, significant collapse of MMP, mitochondrial swelling and finally disruption of mitochondrial outer membrane integrity were occurred. 

 The generation of ROS is also one of the common responses to cellular injury and apoptotic cell death (33). Many studies confirm the generation of various types of reactive oxygen species (ROS) during arsenic metabolism in cells (34). Mitochondria are substantial sources of reactive oxygen species in cell because about 1 -2% of the total oxygen consumed by mitochondria is converted to superoxide anion ( O_2_ ° ^-)^ in mitochondrial respiratory chain at complexes I and III, reduction of ubiquinone, UQ, to its reduced form UQH_2_ proceeds via ubisemiquinone anion radical (UQ-.) (35).As shown on our results, one of the aims in this study was to identify the site of mitochondrial ROS production. Following addition of arsenic into isolated liver mitochondria, rapid increase in H_2_O_2_ formation occurred, which suggests the probable role of mitochondrial H_2_O_2_ in hepatotoxicity associated with arsenic.

 Previous studies showed that Complex I and III are the sources of ROS production in the respiratory chain (15). To distinguish the involvement of complex I and/or III, We measured the rate of both the malate/pyruvate (complex I substrate) - and succinate (complex III substrate)-supported mitochondrial hydrogen peroxide production in liver mitochondria under treatment of different concentrations of arsenic (Figure 2). Our data showed that malate/ pyruvate supports the greatest rate of As (III) induced ROS production but ROS release supported by succinate is not significant in comparison with control group, until in 90 min following As (III) exposure . In addition, malate/pyruvate-supported H_2_O_2_ production in arsenic exposed isolated liver mitochondria is increased significantly by rotenone, an inhibitor of electron transport at the NADH dehydrogenase region, (Figure 2). Increased ROS production with rotenone in succinate fueled isolated mitochondria suggests critical role of NADH-dehydrogenase in arsenicinduced ROS production in liver mitochondria. On the other hand, addition of myxothiazol, an inhibitor of complex III (prevents semiquinone formation) did not show a significant reduction in H_2_O_2_ production in complex III in succinate supported mitochondria. Therefore, our results confirm that complex III is not responsible in arsenic hepatotoxicity.

 Therefore, one of the mechanisms of arsenic induced mitochondrial toxicity is related to the generation of the superoxide anion, which can lead to the formation of more toxic reactive oxygen species, for example, hydrogen peroxide, often taken as the main toxicant due to its mobility and lipid solubility. Therefore, high concentrations of ROS can cause DNA, protein, and lipid damage, as well as changes in antioxidant enzyme expression and cell signal transduction. *In-vitro *studies with endothelial cells suggest that arsenic can cause cellular redox modulation, transcription factor activation, and gene expression relevant to endothelial dysfunction (36). Also; we investigated the effect of arsenic on the activities of complexes II and IV in mitochondrial electron transfer chain. Succinate dehydrogenase (complex II) activity was determined by the reduction of MTT dye to formazone metabolite. Our result showed that arsenic significantly reduced the function of complex II and probably inhibition of this enzyme contributes in arsenic toxicity. On the other hand, arsenic did not have considerable impact on cytochrome c oxidase (complex IV) activity.

Incubation of mitochondrial suspension with arsenic significantly increased lipid peroxidation, which is similar to previous studies in hepatocytes (6, 37). This suggests that mitochondrial lipids are early targets of oxygen free radicals, due to their high content of unsaturated fatty acids and their location in the inner mitochondrial membrane, near the site of ROS production, mainly at the level of complex I and complex III (38). On the other hand, oxidation of lipid membrane results in disruption of mitochondrial membrane and consequently collapse of MMP and cytochrome c release.

In addition to enhanced ROS formation, arsenic also has the ability to complex with -SH groups, thus depleting the level of cellular glutathione (GSH), which plays a critical role in maintaining cellular redox homeostasis (39). On the other hand, it is proven that treatment of liver mitochondria with oxidizing xenobiotics, could decrease GSH levels with the concomitant increase in GSSG concentration. But the GSSG formed was not released from the mitochondria and only reduced back to GSH during recovery of the mitochondria from oxidative stress, unlike the observations reported with isolated hepatocyte, where a rapid efflux of cytosolic GSSG is usually observed following treatment with oxidizing xenobiotics (40). In fact, oxidation of mitochondrial GSH has greater toxic effect on cell viability compared to cytosolic GSH oxidation (41). This is because GSH is not only an antioxidant but also is an essential factor for maintenance of thiol groups of mitochondrial proteins in the reduced state (21). Oxidation of thiol groups from the inner mitochondrial membrane could cause conformational change in mitochondrial permeability transition pore (mPTP) and also MMP, which are generally considered as potential end points in many conditions associated with oxidative stress (42).

Our result suggested that As (III) –induced ΔΨm collapse and apoptosis are associated with thiol oxidation or thiol cross-linkage in MPT pore region. Therefore, decline of reduced glutathione content in mitochondria could cause severe deficiency in their defense system against oxidative damage that leads to the further rise in lipid peroxidative products such as a malondialdehyde (MDA) (2, 43, 44).

Lipid peroxidation not only leads to increase of ROS production but also could damage mitochondrial membrane integrity and open the MPT pores. The opening of the MPT pores is an important step in both necrosis and apoptosis mechanisms (45). Therefore permeabilization of the outer mitochondrial membrane (OMM) and mitochondrial swelling are considered a crucial event during the early phase of apoptosis.

 In our study, the opening of the MPT pores by arsenic is double confirmed by the mitochondrial swelling determination (Table.2) which is subsequent of MMP collapse (Table 1) .This is exactly in accordance with previous studies showed that As (III) caused significant collapse of MMP in treated cells (9, 4, 37).Besides, addition of both Cs A (MPT pore sealing agent) and BHT (ROS scavenger) significantly inhibited the As (III)-induced MMP collapse and mitochondrial swelling (Table1 and 2) suggesting that oxidative stress is directly involved in induction of MPT pore opening. Besides, disruption of mitochondrial outer membrane causes release of mitochondrial cytochrome c, which activates apoptosis process (20). Perier et al reported that cytochrome c is normally bound to the outer surface of the IMM by interactions with mitochondrial phospholipid and can reversibly interact with respiratory chain complexes. It seems that dissociation of cytochrome c from phospholipid such as cardiolipin might be a critical primary step in its release into the cytosol to trigger apoptosome formation (15). On the other hand, Sterndrof *et al*.(1999) realized that As (III) –induced apoptosis was caspase-3 independent and was only partially blocked by a global caspase inhibitor (46). Our results supported that treatment of isolated rat mitochondria with arsenic causes the release of cytochrome c, in order to caspase-3 activation mobilization of cytochrome c from the IMM was not always necessarily involved in As (III) –induced apoptosis.

 The most important function of mitochondria is the generation of adenosine triphosphate (ATP) by oxidative phosphorylation (47). In fact; ATP behaves like a switch between apoptosis and necrosis. Execution of apoptosis needs ATP and depletion of ATP interrupts apoptosis and shifts cell to necrosis (48). Our data showed that incubation of isolated liver mitochondria with As (III) decreased the ATP production and ATP/ADP ratio. This could be resulted from inhibition of mitochondrial respiratory chain and MPT pore opening (15). Opening of MPT pore causes unlimited proton movement across the inner membrane resulted in uncoupling of oxidative phosphorylation and further reduction of ATP concentration leading to exacerbation of ROS production (49). As shown in Figure 7, As (III) significantly increased the outer membrane mitochondrial damage ,another phenomenon which results in cytochrome c release.

 Previous studies revealed that ROS-mediated apoptosis consists MPT pore opening, release of cytochrome c from mitochondria and its ATP dependent interaction with cytosolic factors to activate caspase-3 (37). Therefore, collapse of mitochondrial membrane potential and cytochrome c release are important indicators of cell apoptosis and important endpoints for the determination of mitochondrial dysfunction. Based on our results, As (III) caused significant expulsion of cytochrome c from mitochondria. Besides, Cs A and BHT pretreatment completely blocked arsenic-induced release of cytochrome c from the mitochondria which supports the hypothesis that apoptosis induction by arsenic is due to an oxidative stress dependent opening of the mitochondrial transition pore. On the other hand, arsenic in low concentration could induce apoptosis but probably in higher concentration causes necrosis (due to severe depletion of ATP level). On the other hand, previous studies in human showed that therapeutic dose of As_2_O_3_ (0.16 mg/kg/day) produced 5-10 μM (plasma level) of arsenic without marked toxicity (mild to moderate side effects) which is consistent with the concentrations of As_2_O_3_ needed for MPT pore opening (2). Other clinical study has reported that the plasma levels of arsenic are 5.54–7.30 μM in the patients of acute promyelocytic leukemia treated with As2O3 (50). Hence, mechanisms of toxicity of As_2_O_3_ may be very complex, depending on dose, cell type, or cellular environment (2). In addition, our results suggested that arsenic inhibition of complex I in mitochondrial respiratory chain could induce the uncoupling of electron transport for ATP production, leading to a decrease in mitochondrial membrane potential (Δψ_m_) and subsequent ROS formation. In addition, our results showed that inhibition of complex I by arsenic increased the release of cytochrome c with from mitochondrial inter-membrane space in to cytosolic medium. This can orchestrate the cell death signaling (Figure 8).

**Figure 9:**
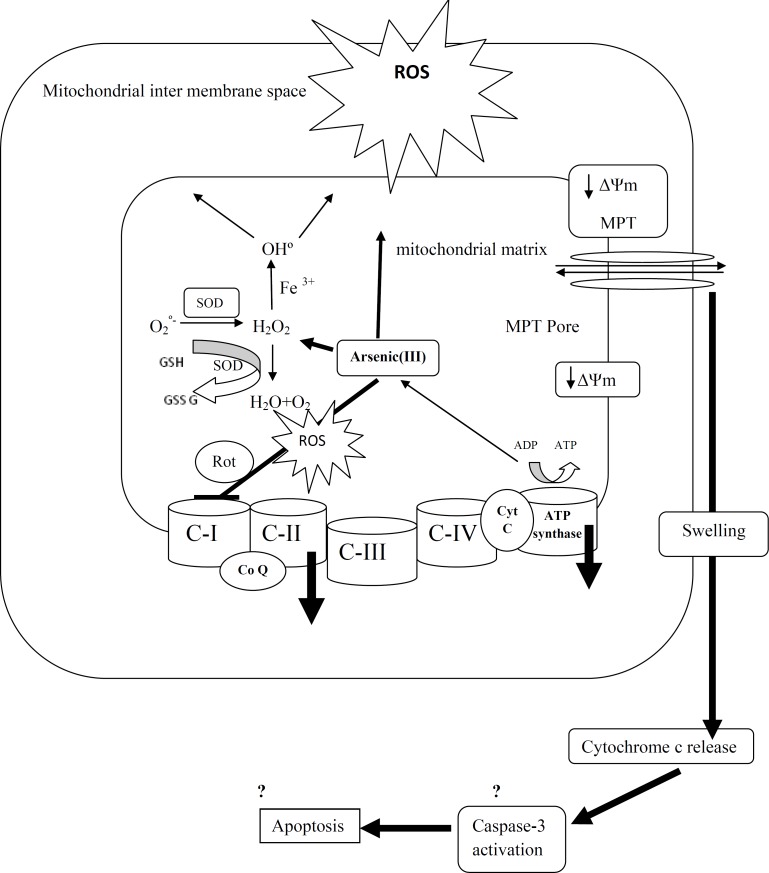
A schematic representation of the mechanisms of arsenic induced release of cytochrome c from mitochondria.

 In conclusion, As (III) impaired the electron transfer chain in isolated mitochondria at complexes I and II which is the cause for increased ROS production. Mitochondrial ROS production contributes in failure of oxidative phosphorylation, decline of cellular ATP, mitochondrial membrane potential disruption, mitochondrial swelling, loss of mitochondrial outer membrane integrity and finally release of cytochrome c which all promote cell death in liver tissue (Figure 9). And finally our research proposed isolated liver mitochondrial as a new model in re-approaching of the etiology of arsenic liver diseases.
